# Personality and Body-Mass-Index in School-Age Children: An Exploration of Mediating and Moderating Variables

**DOI:** 10.1371/journal.pone.0158353

**Published:** 2016-08-03

**Authors:** Mark S. Allen, Stewart A. Vella

**Affiliations:** 1 School of Psychology, Faculty of Social Sciences, University of Wollongong, Northfields Avenue, Wollongong 2522, NSW, Australia; 2 Early Start Research Institute, Faculty of Social Sciences, University of Wollongong, Northfields Avenue, Wollongong 2522, NSW, Australia; University of Calgary, CANADA

## Abstract

This study explored longitudinal associations between personality and body-mass-index (BMI) in school-age children, including the potential mediating role of screen time and physical activity, and the potential moderating roles of child demographics and neighbourhood socioeconomic position. Participants were the parents (and teachers) of 3857 ten-year-old children, who completed questionnaires at baseline with a two-year follow-up. After controlling for child demographics (e.g., sex, pubertal status), we found that personality was unimportant for concurrent BMI, but was important for subsequent BMI and change in BMI over two years. Low levels of introversion and persistence at baseline, and decreases in persistence over time, were associated with a higher BMI at follow-up and a greater increase in BMI over time. Moderator analyses showed that introversion was more strongly related to subsequent BMI for children listed as aboriginal or Torres Strait Islander. The relationship between personality and change in BMI was mediated by screen time, but not by physical activity. To conclude, findings demonstrate that personality is important for change in body mass in Australian children (particularly indigenous children), and that screen-based sedentary behaviour features an important role in this association.

## Introduction

The prevalence of child and adolescent obesity has risen substantially over the past 30 years. In developed countries, 23.8% of boys and 22.6% of girls were overweight or obese in 2013 [1] with indications that increase trends are set to continue. Obesity in childhood has a wide range of serious complications that include an increased risk of premature mortality and physical morbidity in later life [2, 3]. Several factors that relate to diet, physical activity and sedentary behaviour have been found to predict the accumulation of body fat in childhood and adolescence. These include a lack of awareness and false beliefs about nutrition, residence in metropolitan cities, and accessibility to convenience stores and recreational physical activity facilities [4, 5]. In this prospective longitudinal study, we test associations between child personality traits and change in body fat estimates over two years, with a particular focus on demographic and socioeconomic moderators of this association and the potential mediating roles of screen-based sedentary behaviour and physical activity.

Body-mass-index (BMI)–calculated as weight (kg)/height squared (m^2^)–is a useful indirect estimate of total body fat in children and adolescents [6] and is the index most often used to determine body fat. BMI has been found to correlate with personality across the lifespan. In adult samples, longitudinal studies have found that high levels of neuroticism, and low levels of conscientiousness, persistence and novelty seeking, relate to a higher subsequent BMI [7, 8, 9, 10, 11, 12]. In young child samples (~ age 0–8 years), longitudinal studies have found that high levels of emotional reactivity, irritability and impulsivity, and low levels of persistence and self-regulation, relate to a higher subsequent BMI [13, 14, 15, 16, 17]. In school-age children (~ age 6 to 12 years), high levels of introversion, neuroticism and emotionality, and low levels of conscientiousness, relate to a higher BMI [18, 19, 20].

These findings demonstrate that personality is important for BMI across the lifespan, and researchers have begun to explore potential moderators of observed associations. In adult samples, the association between personality and body fat has been found to be moderated by sex, culture and ethnicity [11, 21, 22, 23, 24, 25]. However, findings on moderator analyses have not been consistent across studies [26]. In school-age children, only child sex has been explored as a potential moderator. Pulkki-Råback et al. [19] found that associations between child personality traits and subsequent BMI in adulthood were not moderated by sex, whereas Hampson et al. [18] found that conscientiousness was important for girls’ adult BMI levels and neuroticism was important for boys’ adult BMI levels. Vollrath et al. [20] explored data separately for boys and girls and found similar personality trait associations with BMI across sexes, but also found that personality was more strongly related to being overweight for girls than for boys. In short, more research is needed on potential moderators of personality and body fat associations in school-age children.

Personality is thought to contribute to BMI because individuals with particular personality traits are more or less likely to engage in health-comprising and health-confronting behaviours. Indeed, research in child and adolescent samples has found that personality relates to dietary intake, participation in extracurricular sport, and sedentary behaviours such as television viewing and electronic gaming [13, 20, 27, 28, 29]. There is also considerable evidence that weight management programmes that involve changing unhealthy lifestyle behaviours such as physical inactivity and poor diet contribute to body fat reduction in children (for systematic reviews, see [30, 31, 32, 33]). Together, these findings indicate that personality might relate to the accumulation of body fat through lifestyle behaviours such as diet, physical activity and sedentary behaviour.

Few studies have directly explored the mediating role of health-related behaviours in the association between personality and BMI. In young adults, one study found that physical activity, diet, and eating habits mediated associations between personality (neuroticism, extraversion, openness, and conscientiousness) and BMI [34]. However, dietary intake was not found to mediate a negative association between conscientiousness and BMI in older adults [35]. In a sample of preschool children, enjoyment of food was found to mediate an association between surgency (impulsivity and extraversion) and BMI [17] but the association between surgency and rate of increase in BMI was not mediated by enjoyment of food. In school-age children, associations between child personality traits and eating behaviour were found to align closely with associations between child personality traits and BMI [13] but potential mediating associations were not directly explored. Importantly, one investigation did find that physical inactivity mediated the association between child sociability and subsequent BMI [19]. In short, some initial evidence suggests that an inactive lifestyle mediates the association between personality and BMI in school-age children.

One notable issue from current research on personality and body mass in children is that a variety of traits have been assessed by researchers. The different conceptualisations of personality stem (somewhat) from the different age ranges assessed. To date, researchers have found the development of a general taxonomy of traits for children problematic because child and adolescent maturation leads to an increasingly differentiated set of traits [36]. Nevertheless, child and adolescent traits should be conceptualized with an eye toward adult personality structure [36]. In this study we focus on three traits that are particularly relevant to child behaviour: introversion (the degree to which children enjoy interpersonal interactions), persistence (the degree to which children persevere in challenging conditions) and reactivity (the degree to which children respond emotionally to negative experiences). These traits align closely with three of the big five personality traits commonly explored in adults (extraversion, conscientiousness and neuroticism).

Childhood obesity is related to a variety of serious complications that can impact negatively on health and well-being in later life [2, 3]. Therefore, identifying the factors that contribute to the accumulation of body fat has considerable relevance to public health. There is a shortage of research into the individual characteristics of school-age children and body fat accumulation over time. In this study, we explore the relationship between child personality and concurrent BMI, subsequent BMI, and change in BMI over two years. We further explore four potential moderators–child sex, maturational development (pubertal status), child indigenous status, and neighbourhood socioeconomic position. These variables were explored given their importance in child development [37]. We hypothesised that introversion and reactivity would relate positively, and persistence negatively, to concurrent, subsequent and change in BMI. We further hypothesised that associations would be mediated by physical activity and sedentary behaviour. Moderator analyses were exploratory and no specific hypotheses were generated.

## Method

### Sample

We use data taken from the Longitudinal Study of Australian Children (LSAC) Kindergarten (K) cohort. LSAC aims to investigate social, cultural and environmental influences on child development in Australia. The sample was selected using Medicare Australia’s enrolment database. A random number generator was used to select families from 311 regions (311 postcodes) for potential inclusion in the study. The families of selected children were invited to participate with 54% response rate. Participants provided written informed consent and ethical approval was granted by the Australian Institute of Family Studies ethics committee. Data are collected every 24 months and we use data collected in wave 4 (2010) and wave 5 (2012) that we refer to as Time 1 (all children were age 10) and Time 2 (all children were age 12). In this investigation we use data collected from the child’s school teacher and primary parent (the mother was listed as the primary parent for 97.1% of children). There were 4164 participants at Time 1, of which 307 participants (7.2%) did not return at Time 2. Attrition analyses show that study dropouts had a lower neighbourhood socioeconomic position, *t*(4158) = 3.27, *p* < .01, *d* = 0.19, and a higher BMI, *t*(4008) = 3.59, *p* < .01, *d* = 0.20. Participants that returned at Time 2 were included in this investigation (*n* = 3857). Characteristics of the final sample are reported in Table 1.

**Table 1:** Means, standard deviations, skewness values, and missing cases for study variables at Time 1 and Time 2 (n = 3,857).

	Time 1				Time 2			
	*Mean*	*SD*	*Skew*.	*missing*	*Mean*	*SD*	*Skew*.	*missing*
Neighbourhood socioeconomic position	1013.71	62.99	–1.05	1				
Physical activity	3.29	0.90	0.13	795				
Screen time	2.66	1.50	2.22	2				
Pubertal status	1.69	0.57	–0.17	0	2.13	0.67	–0.92	0
Body-mass-index	18.96	3.71	2.04	135	20.47	3.88	1.18	139
Introversion	2.58	0.77	0.44	33	2.64	0.77	0.37	89
Persistence	3.50	0.89	–0.41	34	3.57	0.86	–0.48	88
Reactivity	2.30	0.81	0.77	33	2.39	0.78	0.68	88

Note: Children are age 10 at Time 1 and age 12 at Time 2. Screen time is in hours per day. Sex, boys *n* = 1970 (51.1%), girls *n* = 1887 (48.9%). Indigenous status, Aboriginal or Torres Strait Islander *n* = 102 (2.6%), non-aboriginal or Torres Strait Islander *n* = 3755 (97.4%).

### Measures

#### Personality

At Time 1 and Time 2, the primary parent completed 12 items from the school aged temperament inventory (SATI [38]) that correspond to three personality dimensions (four items per scale): introversion (e.g., “when meeting new children, acts bashful”; α_T1_ = .79, α_T2_ = .81), persistence (e.g., “does not complete homework unless reminders are given”; α_T1_ = .83, α_T2_ = .81) and reactivity (e.g., “when angry, yells or snaps at others”; α_T1_ = .88, α_T2_ = .87). Parents were given the following instructions: “Please select the response that best describes how often your child’s behaviour matches the behaviour described in the statement”. Items on the SATI are scored on a 5-point scale from 1 (*never*) to 5 (*always*) with higher scores indicative of greater introversion, persistence and reactivity. The SATI has demonstrated high test-retest reliability for parent-report assessments [38], but has not been subjected to critical confirmatory (construct, concurrent) validation.

#### Body-mass-index

At Time 1 and Time 2, height and weight were measured directly by a trained professional. Weight was measured using Tanita body fat scales and height was measured using a laser stadiometer. For height, two measurements were taken, and if the two measurements differed by more than 0.5 cm a third measurement was taken, with the average of the two closest measurements recorded as the child’s height. Height and weight were used to calculate BMI (kg/m^2^). The use of raw BMI scores for data analysis, rather than BMI z scores, is recommended for longitudinal designs, particularly when the paediatric sample do not vary in age [39, 40, 41, 42].

#### Physical activity and sedentary behaviour

Teacher reported child physical activity was taken at Time 1 using the mean of two items: “during organised activities for your class, how does this child compare with other children in the class in terms of level of physical activity?” and “during play with friends at recess or lunch time, how does this child compare with other children in the class in terms of level of physical activity?”. Each item was reported on a scale from 1 (*a lot less active than most*) to 5 (*a lot more active than most*). At Time 1, the primary parent also reported the number of minutes the child spends watching television and playing electronic games on an average week day and on an average weekend day. Values were weighted (weekday x 5, and weekend day x 2) to provide an estimate of minutes spent television viewing and electronic gaming in an average week. We calculated a composite score for the two screen-based activities (total screen time) and recoded from minutes per week into hours per day to aid interpretation of the unstandardized regression coefficients.

#### Control variables

Parents listed their postcode and provided information on child sex, indigenous status, and pubertal status at Time 1. Pubertal status was measured at Time 1 using three items for boys (growth spurt, body hair, skin changes) and an additional fourth item (breast growth) for girls. At Time 2, two additional items were included for boys (deepening of voice, hair growth on face). Items were adapted from the pubertal development scale for parental report [43] and were assessed from 1 (*has not started yet*) to 4 (*seems complete*). Using participants’ postcode, an estimate of neighbourhood socioeconomic position (NSP) was determined according to the Index of Relative Socio-Economic Disadvantage [44].

### Data Analyses

Consistent with previous research [7] we tested three linear regression models that correspond to cross-sectional, longitudinal, and change associations. For cross-sectional associations (Model 1), we regressed Time 1 BMI on Time 1 personality traits (introversion, persistence, and reactivity) and four control variables measured at Time 1 (child sex, indigenous status, NSP and pubertal status). For longitudinal associations (Model 2), we regressed Time 2 BMI on Time 1 personality traits (introversion, persistence, and reactivity), the change in personality traits between Time 1 and Time 2 (Time 1 score subtracted from Time 2 score for introversion, persistence, and reactivity) and five control variables: Time 1 assessments of child sex, indigenous status, NSP and pubertal status, and the change in pubertal status between Time 1 and Time 2. For change associations (Model 3), we conducted the same regression model as Model 2 with Time 1 BMI included as an additional control variable.

We subsequently explored the moderating role of control variables (sex, pubertal status, indigenous status, and NSP) in cross-sectional, longitudinal, and change associations. Twelve interaction terms were computed from standardised data [45]–the four control variables as moderators of the three personality dimensions measured at baseline–and each moderator (three interaction terms) was explored independently. We further explored whether change associations were mediated by physical activity or screen time. In three independent models, one personality trait (baseline measure) was set as the independent variable, physical activity and screen time were set as mediating variables, Time 2 BMI was set as the dependent variable, and Time 1 BMI was set as a control variable. We controlled for sex, indigenous status, NSP, pubertal status and change in pubertal status in each model. A bootstrapping procedure was used to estimate indirect paths [46]. The bootstrapping procedure involved 5000 resamples and the meaningfulness of the indirect paths was determined according to bias corrected and accelerated (BCA) 95% confidence intervals [46].

## Results

Means, standard deviations, skewness values, and missing cases for study variables at Time 1 and Time 2 are presented in Table 1. Between age 10 and age 12, there were small increases in introversion, *t*(3543) = 5.89, mean diff. = .06 [BCA 95% CI: .04, .08], *d* = 0.08, persistence, *t*(3543) = 5.96, mean diff. = .07 [BCA 95% CI: .05, .10], *d* = 0.08, and reactivity, *t*(3543) = 7.89, mean diff. = .09 [BCA 95% CI: .07, .11], *d* = 0.11, and a medium increase in BMI, *t*(3543) = 44.77, mean diff. = 1.53 [BCA 95% CI: 1.47, 1.59], *d* = 0.42.

### Main effects

Findings from the linear regression models are presented in Table 2. For Model 1, a higher BMI at baseline was associated with a lower neighbourhood socioeconomic position (*b* = –.05, *s*.*e*. = .01) and a higher pubertal status (*b* = 1.53, *s*.*e*. = .13). Baseline BMI was also higher in girls than in boys (*b* = –.78, *s*.*e*. = .15). The personality traits of introversion, persistence and reactivity were unrelated to concurrently measured BMI. For Model 2, all demographic and socioeconomic control variables were associated with follow-up measures of BMI. Children with a lower neighbourhood socioeconomic position (*b* = –.07, *s*.*e*. = .01), a higher pubertal status (*b* = 2.20, *s*.*e*. = .15), and a greater increase in pubertal status over time (*b* = .72, *s*.*e*. = .13) had a higher BMI at follow-up. A higher BMI at follow-up was also observed for girls (*b* = –.56, *s*.*e*. = .16) and for children listed as aboriginal or Torres Strait Islander (*b* = 1.46, *s*.*e*. = .41). Lower levels of introversion (*b* = –.35, *s*.*e*. = .09) and persistence (*b* = –.32, *s*.*e*. = .09) at baseline were associated with a higher BMI at follow-up, and a greater increase in persistence over time (*b* = –.21, *s*.*e*. = .10) was associated with a lower BMI at follow-up.

**Table 2:** Linear regression models for personality on BMI.

	1. Baseline BMI			2. Follow-up BMI			3. Follow-up BMI		
	*b* (s.e.)	˅ 95% CI	˄ 95% CI	*b* (s.e.)	˅ 95% CI	˄ 95% CI	*b* (s.e.)	˅ 95% CI	˄ 95% CI
Baseline BMI	-	-	-	-	-		.90 (.01)***	.84	.97
Sex	–.78 (.15)***	–1.10	–.47	–.56 (.16)***	–.87	–.24	.33 (.09)***	.11	.54
Indigenous status	.59 (.40)	–.48	1.76	1.46 (.41)***	.34	2.56	.52 (.23)*	–.07	1.06
NSP	–.05 (.01)***	–.07	–.03	–.07 (.01)***	–.09	–.05	–.02 (.01)***	–.03	–.01
Pubertal status	1.53 (.13)***	1.27	1.80	2.20 (.15)***	1.90	2.52	.52 (.08)***	.33	.71
Δ Pubertal status	-	-	-	.72 (.13)***	.47	.99	.26 (.07)***	.09	.44
Introversion	–.12 (.08)	–.28	.04	–.35 (.09)***	–.53	–.19	–.12 (.05)*	–.21	–.03
Persistence	–.08 (.08)	–.24	.06	–.32 (.09)***	–.51	–.12	–.21 (.05)***	–.30	–.14
Reactivity	.05 (.08)	–.11	.19	.11 (.10)	–.09	.31	.09 (.05)	–.01	.18
Δ Introversion	-	-	-	–.18 (.11)	–.41	.06	–.03 (.06)	–.14	.09
Δ Persistence	-	-	-	–.21 (.10)*	–.42	.00	–.16 (.06)**	–.26	–.07
Δ Reactivity	-	-	-	.20 (.11)	–.02	.46	.11 (.06)	.02	.22
Explained variance (*R*^2^)	.047***			.089***			.732***		

Note: BMI, body-mass-index. Δ, change score. NSP, neighbourhood socioeconomic position. Bias corrected and accelerated 95% confidence intervals reported (1000 resamples for bootstrap). Missing cases were not MCAR, χ^2^(157) = 376.31, *p* < .001, and therefore were handled through listwise deletion. Model 1, *n* = 3698, Model 2, *n* = 3646, Model 3, *n* = 3543. Missing data analyses (Model 3) show that excluded participants had a higher BMI at Time 1, *t*(3720) = 7.17, *p* < .001 and Time 2, *t*(3716) = 5.13, *p* < .001, a lower NSP, *t*(3854) = 6.58, *p* < .001, higher reactivity at Time 1, *t*(3822) = 2.78, *p* < .01, and Time 2, *t*(3767) = 2.09, *p* < .05, lower persistence at Time 2, *t*(3767) = 1.98, *p* < .05, and a lower pubertal status at Time 2, *t*(3855) = 12.05, *p* < .001.

*p < .05

**p < .01

***p < .001.

For Model 3, all demographic and socioeconomic control variables were associated with change in BMI over time. Children with a lower neighbourhood socioeconomic position (*b* = –.02, *s*.*e*. = .01), a higher pubertal status (*b* = .52, *s*.*e*. = .07), and a greater increase in pubertal status over time (*b* = .26, *s*.*e*. = .07) had a greater increase in BMI. A greater increase in BMI was also observed for boys (*b* = .33, *s*.*e*. = .09) and for children listed as aboriginal or Torres Strait Islander (*b* = .52, *s*.*e*. = .23). Children with lower levels of introversion (*b* = –.12, *s*.*e*. = .05) and persistence (*b* = –.21, *s*.*e*. = .05) at baseline also had a greater increase in BMI over time, and a decrease in persistence over time (*b* = –.16, *s*.*e*. = .06) was associated with an increase in BMI over time. Taken together, the linear regression models demonstrate that child personality traits are important for subsequent BMI and change in BMI over two years, but are unimportant for concurrent BMI.

### Moderation

For Model 1, the association between personality and concurrent BMI was not moderated by child sex, indigenous status, pubertal status, or NSP. For Model 2, there was a significant moderation by child indigenous status for introversion on subsequent BMI (*b*_*interaction*_ = –1.31, *s*.*e*. = .41 [BCA 95% CI: –2.34,–.28]), demonstrating a stronger association between introversion and subsequent BMI for children listed as aboriginal or Torres Strait Islander. The association between personality and subsequent BMI was not moderated by child sex, pubertal status or NSP. For Model 3, the association between personality and change in BMI was not moderated by child sex, indigenous status, pubertal status, or NSP.

### Mediation

Findings from the multiple mediator models are presented in Table 3. There was a significant effect for introversion (*b* = –.09, *s*.*e*. = .02), persistence (*b* = .16, *s*.*e*. = .02) and reactivity (*b* = –.10, *s*.*e*. = .02) on physical activity levels, and a significant effect for persistence (*b* = –.19, *s*.*e*. = .03) and reactivity (*b* = .13, *s*.*e*. = .03) on screen time. Screen time, but not physical activity, was associated with change in BMI in all three models (Table 3, b paths). There was a significant effect for persistence (*b* = –.18, *s*.*e*. = .04) and reactivity (*b* = .13, *s*.*e*. = .05) on change in BMI. Bootstrap results for indirect effects showed a mediating effect for screen time in the association between persistence and change in BMI (Δ*b* = –.022, BCA 95% CI:–.040,–.010) and a mediating effect for screen time in the association between reactivity and change in BMI (Δ*b* = .016, BCA 95% CI: .006, .034). The upper- and lower-bound confidence intervals were outside of zero indicating that these mediation effects were meaningful.

**Table 3:** Summary of multiple mediator models for personality on BMI through physical activity and screen time.

	Introversion	Persistence	Reactivity
a paths (IV to mediators)			
Physical activity	–.09 (.02)***	.16 (.02)***	–.10 (.02)***
Screen Time	.04 (.03)	–.19 (.03)***	.13 (.03)***
b paths (mediators to DV)			
Physical activity	–.03 (.04)	.00 (.04)	–.01 (.04)
Screen Time	.12 (.03)***	.11 (.03)***	.12 (.03)***
c path (IV to DV, total effect)			
BMI	–.07 (.05)	–.18 (.04)***	.13 (.05)**
c' path (IV to DV, direct effect)			
BMI	–.07 (.05)	–.16 (.04)***	.12 (.05)
Partial effect of control variables on DV			
Baseline BMI	.89 (.01)***	.89 (.01)***	.89 (.01)***
Sex	.26 (.09)**	.32 (.09)***	.26 (.09)**
Indigenous status	.68 (.24)**	.63 (.24)**	.65 (.24)**
Neighbourhood socioeconomic position	–.02 (.01)**	–.02 (.01)**	–.02 (.01)**
Pubertal status	.61 (.09)***	.60 (.09)***	.60 (.09)***
Δ Pubertal status	.26 (.07)***	.26 (.07)***	.25 (.07)***
Bootstrap results (bias corrected and accelerated 95% CI’s)			
Physical activity	.002 [–.005, .012]	.000 [–.015, .015]	.001 [–.008, .011]
Screen Time	.005 [–.002, .017]	–.022 [–.040,–.010]	.016 [.006, .034]
Explained variance (*R*^2^)	.73***	.73***	.73***

Note: BMI, body-mass-index. Δ, change score. Missing data were handled through listwise deletion. Final models, *n* = 2,870. Missing data analyses show that excluded participants had a higher BMI at Time 1, *t*(3720) = 4.29, *p* < .001, and Time 2, *t*(3716) = 2.15, *p* < .05, a lower NSP, *t*(3854) = 3.37, *p* < .01, lower physical activity, *t*(3060) = 2.13, *p* < .05, lower persistence at Time 1, *t*(3821) = 4.76, *p* < .001, and Time 2, *t*(3767) = 4.03, *p* < .001, higher reactivity at Time 1, *t*(3822) = 2.75, *p* < .01, and a lower pubertal status at Time 2, *t*(3855) = 3.84, *p* < .001.

**p < .01

***p < .001.

## Discussion

This prospective longitudinal study tested associations between child personality and change in body fat estimates over two years, including the assessment of demographic and socioeconomic moderators of this association and the potential mediating roles of screen-based sedentary behaviour and physical activity. Personality traits were unimportant for concurrent BMI, but were important for subsequent BMI and change in BMI. In particular, low levels of introversion and persistence at baseline, and decreases in persistence over time, were associated with a higher BMI at follow-up and a greater increase in BMI over time. Moderator analyses demonstrated that the negative association between introversion and subsequent BMI was stronger for indigenous Australian children. Mediation analyses demonstrated that personality was related to change in BMI through the variance shared with sedentary behaviour.

The finding that introversion and persistence were unrelated to concurrent BMI, but were important for subsequent BMI and change in BMI is intriguing and somewhat consistent with what has been observed in previous studies of school-age children. Indeed, cross-sectional studies have often reported no association between personality and BMI [20, 47], with prospective longitudinal studies showing significant associations [18, 19]. Our findings demonstrate that lower levels of introversion and lower levels of persistence at age 10 are associated with a higher BMI at age 12 and a greater increase in BMI between age 10 and age 12. Also, that decreases in persistence between age 10 and age 12 coincide with increases in BMI between age 10 and age 12. The direction of these associations is not entirely consistent with study hypotheses. As hypothesised, lower levels of persistence were related to a higher BMI, but rather than high levels of introversion relating to a higher BMI, our findings demonstrate that low levels of introversion relate to a higher BMI. Previous research on the relationship between introversion and BMI has been mixed. Some prospective studies indicate no association [18] and others indicate a positive association [19]. However, differences in study designs, and in particular the timeframes between measures of personality and BMI, mean that direct comparisons are problematic. Clearly more research is warranted. Nevertheless, in the current sample it would appear that more sociable (extraverted) children are at a greater risk of increased body fat over time.

There was little evidence that child demographics moderate associations between personality and BMI. In previous studies, only child sex has been explored as a potential moderator in school-age children, with little evidence that associations differ between boys and girls [18, 19]–although sex appears an important moderator in adult samples [25]. Our findings show that associations between personality and BMI are not moderated by child sex. We also explored the potential moderating role of maturational development (pubertal status), indigenous status, and neighbourhood socioeconomic position. There were no moderation effects for pubertal status or neighbourhood socioeconomic position, but introversion was more strongly related to subsequent BMI for children listed as aboriginal or Torres Strait Islander. This finding complements new studies in adult samples that have implicated ethnicity as an important moderator [11, 25, 26]. It would appear that more sociable (extraverted) 10-year-old children are at greater risk of a higher BMI at age 12 if they are aboriginal or Torres Strait Islander.

Studies have started to explore behaviours that might mediate associations between personality and BMI, with enjoyment of food [17] and physical inactivity [19] identified as important mediators in childhood samples. Our findings show that teacher-reported school-based physical activity did not mediate associations between personality and BMI, but parent-reported screen-based sedentary behaviour (television viewing and electronic gaming) mediated a negative association between persistence and change in BMI and a positive association between reactivity and change in BMI. Children with lover levels of persistence or higher levels of reactivity were more sedentary (in terms of total screen time) and children that were more sedentary had a higher BMI. We are unable to make causal inferences from the association data reported, but these findings suggest that targeting a reduction of screen time (rather than increasing physical activity), might be a useful approach to preventing the accumulation of body fat in children with personality traits associated with a high risk of body fat accumulation.

Strengths of this investigation include the objective assessment of child BMI, the large nationally-representative sample, and the longitudinal nature of the data. However there are also some important limitations that readers must consider in their interpretation of study findings. First, the assessment of physical activity included only within-school activity levels as perceived by the child’s teacher, and the assessment of sedentary behaviour was parent report and did not include non-screen-based sedentary behaviour (e.g., sitting down at school). More objective measures of physical activity and sedentary behaviour might provide more accurate estimates of effect sizes for mediation models than those reported in the current investigation. Second, missing data analyses show that study dropouts and excluded participants had a higher BMI, higher scores for reactivity and lower scores for persistence, potentially weakening observed associations. Third, that multiple tests were conducted to explore moderator variables increases the probability of a Type I error. Replication studies are required to test further the meaningfulness of the ethnicity moderation effect identified in this study. Fourth, the measure of body composition used in the study (BMI) provides an indirect estimate of child adiposity and other measures (e.g., the body adiposity index) might provide more accurate estimates (see, for example [48]) and offer more accurate effect size estimates for associations with personality. Further experimental research is needed to identify whether changing health-related behaviour can affect BMI for children with particular personality characteristics.

## Conclusion

This study explored associations between child personality and change in body fat estimates over two years, including potential demographic and socioeconomic moderators and the potential mediating roles of screen-based sedentary behaviour and physical activity. Our findings show that more sociable and less persistent 10-year-old children have an increased risk of higher body fat at age 12, and that children who increase the trait of persistence between age 10 and age 12 tend to decrease body fat between age 10 and age 12. Importantly, the negative association between introversion and subsequent BMI was stronger for children listed as aboriginal or Torres Strait Islander and personality traits were related to BMI, in part, through the variance shared with screen-based sedentary behaviour. These findings (should they be replicated) might be of value to health consultants targeting the reduction of body fat in children. Personality tests might offer a useful approach to identifying children at greater risk of excessive sedentary behaviour and in turn a higher BMI, particularly among indigenous Australian populations. In addition to identifying ‘at risk’ populations, these findings might also be of interest to parents and general practitioners. Limiting the number of hours children spend in screen-based activities, rather than increasing physical activity, might be a more effective approach to preventing the accumulation of excessive body fat. We recommend replication studies that directly address study limitations and experimental research targeting the reduction of screen-based sedentary behaviour and body fat in children.
